# Gastrointestinal stromal tumor with small cell carcinoma infiltration: a case report

**DOI:** 10.3389/fonc.2024.1389975

**Published:** 2024-06-17

**Authors:** Yitong Zhou, Liyuan Song, Li Lyu, Shengjie Li, Qimin Wang

**Affiliations:** Department of Pathology, The Second Affiliated Hospital of Dalian Medical University, Dalian, Liaoning, China

**Keywords:** gastrointestinal stromal tumors, small cell carcinoma, small cell lung cancer (SCLC), neoplasm metastasis, case report

## Abstract

Gastrointestinal stromal tumors (GISTs) are the most common mesenchymal tumors of the digestive system. They usually occur in the gastrointestinal tract. However, we discovered a rare phenomenon in which small cell carcinoma infiltrated the GIST of a patient. The patient came to the hospital and presented with chest tightness and shortness of breath for 2 months and a dry cough for half a month. As the ancillary tests were refined, it was discovered that he also had a lesion in the pelvic cavity. After pathological examination of the core needle biopsy (CNB) samples from the pelvic cavity lesion, the patient was diagnosed with GIST with small cell carcinoma infiltration. The patient is currently receiving a chemotherapy regimen of etoposide combined with cisplatin.

## 1 Introduction

Gastrointestinal stromal tumors (GISTs) are the most common mesenchymal tumors of the digestive system (1). The histological appearance of GISTs is mainly characterized by spindle-shaped cells (2). Morphologically, epithelioid cells, palisaded–vacuolated cells, and sclerotic patterns may also be present (2). GISTs generally express positive indicators such as CD117 and DOG1 according to immunohistochemistry (IHC) staining (3).

Small cell carcinoma is a type of neuroendocrine carcinoma. When small cell carcinoma occurs in the lung, it can also be known as small cell lung cancer (SCLC). SCLC accounts for approximately 15% of all lung cancers (4). In SCLC, finely granular nuclear chromatin, absent nucleoli, and common fusiform shapes are histologically found, and the nuclear/cytoplasmic ratio is generally high (5). In terms of IHC staining, SCLC is usually positive for thyroid transcription factor-1 (TTF-1), cytokeratin (CK), and synaptophysin (Syn) (6, 7).

SCLC commonly metastasizes to the brain, bones, liver, and adrenal glands (8), but the metastasis of SCLC to other solid tumors is not commonly reported. GISTs have been reported to accompany SCLC (9). However, small cell carcinoma infiltrating GISTs has not been found.

## 2 Case description

### 2.1 Clinical characteristics

A 69-year-old male patient with a 10-year history of arterial hypertension presented to the Medical Oncology Outpatient with chest tightness and shortness of breath for 2 months and a dry cough for half a month. A chest CT revealed an infection in the upper lobe of the left lung and a lesion in the left hilar region. After antibiotic treatment, the patient underwent a repeat chest CT scan, which revealed no significant changes in the mediastinum or left hilar mass. On admission, he underwent a chest contrast-enhanced CT, which revealed a lesion in the left hilar region measuring approximately 43 × 63 mm and encroaching on the left pulmonary artery, suggesting malignancy. The patient then underwent a CT-guided percutaneous core needle biopsy for the left hilar lesion. The patient’s left lung biopsy tissue was sent for pathological examination. The contrast-enhanced CT of the abdomen revealed a hepatic cyst. Pelvic cavity CT revealed a pelvic cavity soft tissue lesion, approximately 61 × 63 mm in size.

**Figure 1:**
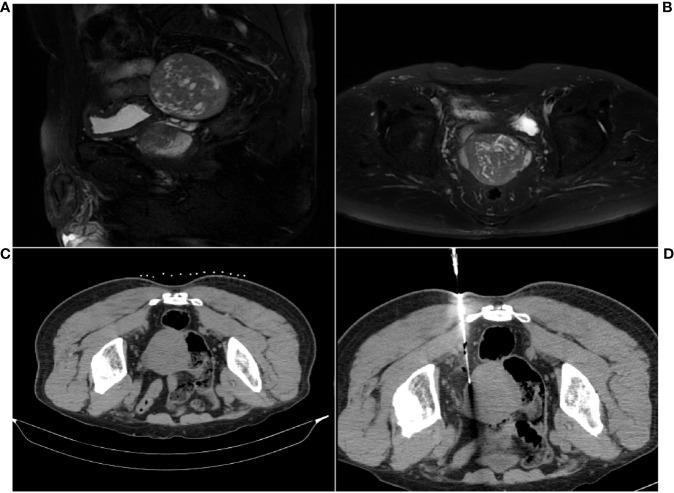
MRI and CT scan images of the pelvic cavity lesion. **(A)** Sagittal MR image. **(B)** Axial MR image. **(C)** Well-defined roundish mass. **(D)** CT scan during the core needle biopsy for sample collection.

In response to the discovery of the pelvic cavity lesion, the patient underwent additional contrast-enhanced MRI, which revealed a posterior bladder lesion (**Figures 1A, B**). It presented a well-defined roundish mass, mostly with slightly low and uneven signals, suggesting possible malignancy. Subsequently, the patient underwent a CT-guided percutaneous core needle biopsy for the pelvic cavity lesion. CT revealed a mostly well-defined roundish mass. As expected, hypodense lesions were observed in a small portion of the ill-defined part of the mass (**Figure 1C**). Clinicians then chose a core needle biopsy site that allows obtaining lesions of different densities at the same time (**Figure 1D**). The biopsy tissue was sent for pathological examination.

### 2.2 Histological characteristics and IHC staining

**Figure 2:**
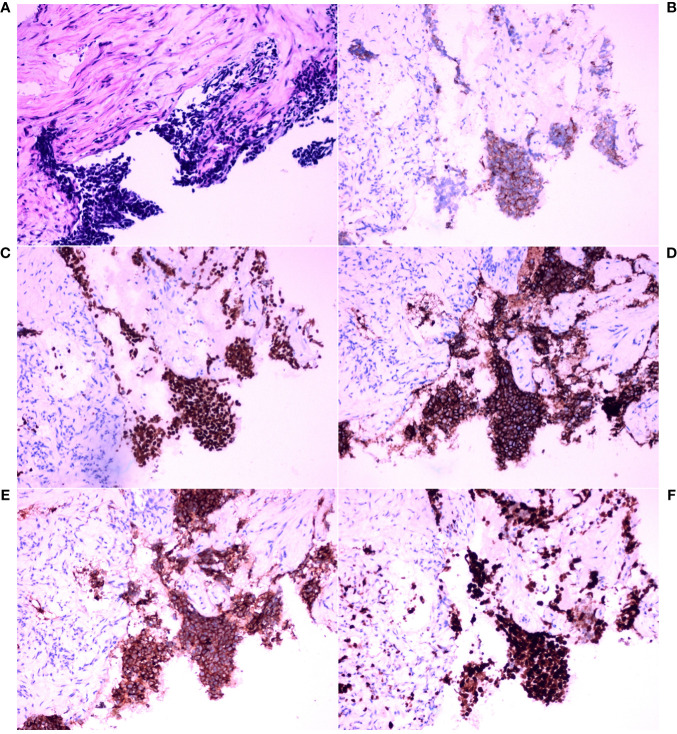
Histopathological analysis and immunohistochemical examination of the core needle biopsy tissue in the left hilar tumor. **(A)** Hematoxylin and eosin (HE) staining (×200). **(B)** Immunohistochemical (IHC) staining for cytokeratin (CK) (×200). **(C)** IHC staining for thyroid transcription factor-1 (TTF-1) (×200). **(D)** IHC staining for CD56 (×200). **(E)** IHC staining for synaptophysin (Syn) (×200). **(F)** IHC staining for Ki-67 (×200).

The left hilar biopsy tissue was susceptible to stretching and deformation, and analysis of the morphological features revealed nests, beams, and chrysanthemum-shaped clusters of arranged cells. The tumor cells were small, with little cytoplasm, indistinct cell boundaries, fine chromatin, and a lack of nucleoli (**Figure 2A**). IHC staining revealed that the tumor cells were positive for CK, TTF-1, CD56, and Syn, and the Ki-67 index was approximately 90%. Ultimately, the IHC staining results and morphological features were combined to pathologically diagnose poorly differentiated neuroendocrine carcinoma (small cell carcinoma) (**Figures 2B–F**).

**Figure 3:**
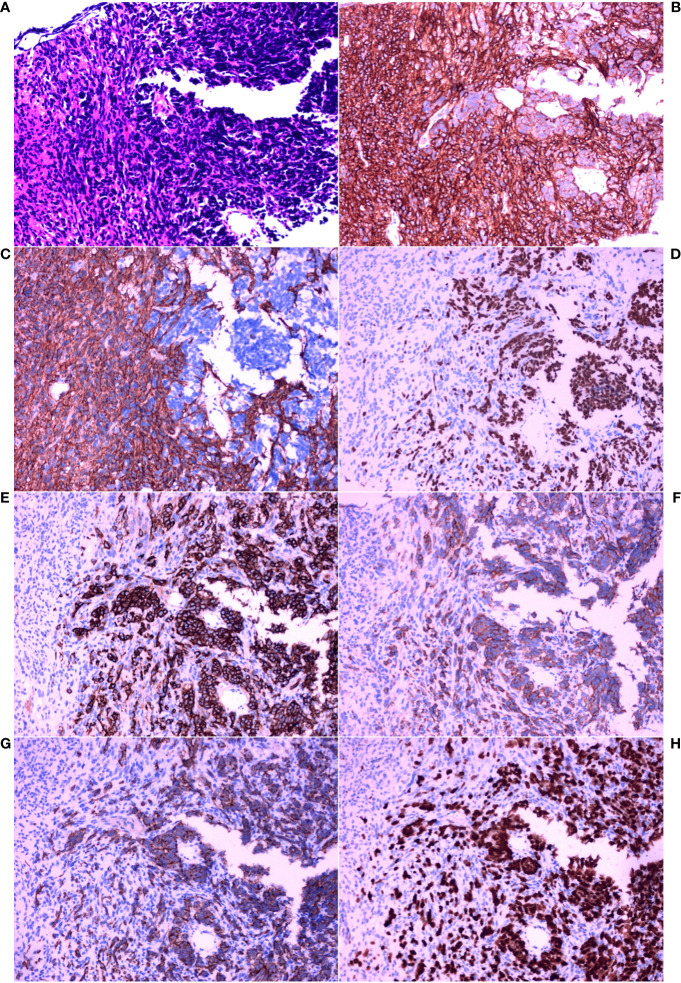
Histopathological analysis and immunohistochemical examination of the pelvic cavity lesion sample obtained by core needle biopsy. **(A)** HE staining (×200). **(B)** IHC staining for CD117 (×200). **(C)** IHC staining for DOG1 (×200). **(D)** IHC staining for thyroid transcription factor-1 (TTF-1) (×200). **(E)** IHC staining for CD56 (×200). **(F)** IHC staining for synaptophysin (Syn) (×200). **(G)** IHC staining for cytokeratin (CK) (×200). **(H)** IHC staining for Ki-67 (×200).

**Figure 4:**
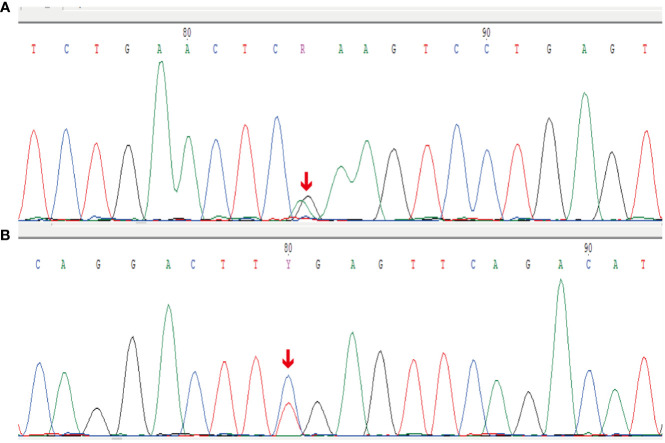
Sanger sequencing results for the c-kit gene. **(A)** Forward reads from exon 13 c.1924A>G (p.K642E) gain-of-function mutation of the c-kit gene. **(B)** Reverse reads from exon 13 c.1924A>G (p.K642E) gain-of-function mutation of the c-kit gene.

Thereafter, morphological analysis of the biopsy tissue from the pelvic cavity lesion revealed that some areas presented a chrysanthemum cluster-like structure, consisting of spindle cells and epithelioid cells, with visible paranuclear vacuoles (**Figure 3A**). These histological features were consistent with the diagnosis of GIST. Immunohistochemical staining of the spindle and epithelioid cell tumor region revealed that the tumor was positive for CD117 and DOG1 (**Figures 3B, C**), but negative for smooth muscle actin (SMA), CD34, S-100, CD10, HMB45, and desmin. Genetic testing confirmed the diagnosis of GIST, and a gain-of-function mutation was identified in exon 13 c.1924A>G (p.K642E) of the c-kit gene by Sanger sequencing (**Figure 4**).

However, between these spindle-shaped and epithelioid cells, there were some basal cells arranged in a fenestrated pattern. These infiltrating cells were small, round or short, shuttle-shaped, with a naked nucleus, deep nuclear staining, and inconspicuous nucleoli. These features were similar to those of small cell carcinoma.

Therefore, additional targeted IHC staining of the biopsy tissue from the pelvic cavity lesion was carried out. Infiltration of small cell carcinoma in the GIST was found (**Figure 3A**). The infiltrating fraction expressed TTF-1, CD56, Syn, and CK and had a proliferation index of approximately 90%, as measured using Ki67 IHC staining (**Figures 3D–H**). Considering these findings and the morphological features, it was concluded that the lesion in the pelvic cavity was GIST with small cell carcinoma infiltration. Fortunately, there was no evidence that the SCLC had metastasized to any site other than the pelvic cavity.

### 2.3 Treatment after diagnosis

The patient first underwent treatment with etoposide combined with cisplatin for 21 days per cycle. After three cycles of chemotherapy, the patient’s GIST had ruptured and bled, necessitating surgery. Postoperative histopathology revealed necrosis in the area infiltrated by small cell carcinoma. The patient is still undergoing chemotherapy for small cell carcinoma of the lungs. Postoperative histopathology of the GIST revealed that the current chemotherapy regimens are effective.

## 3 Discussion

SCLC often metastasizes to the bone and the brain in advanced stages (10). Metastasis to other sites is rare. Only a few studies have reported GISTs with neuroendocrine carcinoma components. Phan et al. (9) reported on a patient with pancreatic GIST and limited-stage SCLC. However, this patient’s GIST and SCLC occurred in two different lesion tissues. Our patient suffered from advanced-stage SCLC. The pathological features of our patient corroborate the aggressiveness of small cell carcinoma: it may invade less malignant tumors. A patient with simultaneous poorly differentiated neuroendocrine carcinoma and GIST of the stomach was identified by Ding et al. (11). These authors explained this phenomenon by the fact that GISTs and neuroendocrine tumors (NETs) have some commonalities in their immunoreactivity profiles, which could indicate a common oncogenic mechanism for neuroendocrine cancer and GISTs (11). Their cases had a histological pattern typical of GIST. Moreover, the expression of IHC in the neuroendocrine carcinoma was similar to that of our patient. The difference was that, in their cases, the neuroendocrine carcinoma did not invade the GIST. Our case likely presented metastasis from the primary tumor in the left lung, which led to the GIST being infiltrated by small cell carcinoma.

The literature shows that GIST and neuroendocrine carcinoma have some common immunophenotypes. CD117 and DOG1 positivity is commonly used clinically to aid in the diagnosis of GIST (12). However, Akintola-Ogunremi et al. (13) demonstrated that CD117 is expressed in some neuroendocrine carcinomas and has potential therapeutic implications. Marando et al. (14) reported a statistically significant correlation between DOG1 expression and neuroendocrine tumors of the gastrointestinal tract. This evidence may suggest that it is not a coincidence that our case of GIST was infiltrated by small cell neuroendocrine carcinoma. GISTs could also coexist with a single well-differentiated, sporadic, small NET (15).

The metastasis and infiltration of SCLC into the GIST in our patient may not have been coincidental. Thus, the potential underlying mechanism was explored based on previous studies. An *in vivo* experiment on SCLC showed that tumors are generally composed of phenotypically distinct cells characterized by neuroendocrine or mesenchymal markers, and these cells have a common origin (16). The biological behavior of tumor cells is affected by the crosstalk between mesenchymal and neuroendocrine cells. Mesenchymal cells can confer metastatic capacity on neuroendocrine cells (16). GISTs are tumors derived from mesenchymal cells. This might represent a potential connection. Genetically, GIST and SCLC share some commonly mutated genes, such as *FGFR1* and *KIT* (17, 18). The diseases also share abnormal expression of some molecules in common pathways such as PI3K/AKT and MAPK (19–21).

Moreover, SCLC is highly vascularized, and angiogenesis greatly contributes to its metastatic process (22). Small cell carcinomas could metastasize via the bloodstream with a tumor-homing effect and are more likely to grow in the setting of gastrointestinal mesenchymal tumors. Epithelial-to-mesenchymal transition (EMT) could also play an important role in the metastasis of small cell carcinoma. A study of drug responses in small cell carcinoma revealed that it partly exhibits features of EMT (23). In this process, small cell carcinomas may express chemokines that target the GIST. The molecular and cellular mechanisms of small cell carcinoma homing and EMT in specific environments are poorly understood.

Exploring these mechanisms could lead to the design of better treatments for patients with poorly differentiated small cell carcinoma. However, our case is an isolated one. Our findings do not rule out the possibility that these tumors occurred coincidentally. More experiments are needed to ascertain the link between the two diseases. The patient had been treated with chemotherapy for SCLC for several months prior to undergoing resection of the GIST. This may have led to necrosis in the area infiltrated by the small cell carcinoma in the GIST, causing the GIST to rupture. Hence, the small cell carcinoma infiltrating area became a range of necrosis in the resection specimens. It is difficult to explore the mechanisms in resection specimens.

## Data availability statement

The original contributions presented in the study are included in the article/supplementary material. Further inquiries can be directed to the corresponding authors.

## Ethics statement

The studies involving humans were approved by Ethics Committee of the Second Affiliated Hospital of Dalian Medical University. The studies were conducted in accordance with the local legislation and institutional requirements. The human samples used in this study were acquired from a by-product of routine care or industry. Written informed consent for participation was not required from the participants or the participants’ legal guardians/next of kin in accordance with the national legislation and institutional requirements. Written informed consent was obtained from the individual(s) for the publication of any potentially identifiable images or data included in this article.

## Author contributions

YZ: Conceptualization, Data curation, Formal analysis, Investigation, Methodology, Software, Writing – original draft, Writing – review & editing. LS: Supervision, Validation, Visualization, Writing – review & editing. LL: Conceptualization, Resources, Validation, Writing – review & editing. SL: Conceptualization, Resources, Validation, Writing – review & editing. QW: Conceptualization, Methodology, Project administration, Resources, Supervision, Validation, Writing – review & editing.
